# Multi-DDA: drug–disease association prediction using a hybrid graph convolutional network with multi-modal drug representations

**DOI:** 10.1093/bioadv/vbag034

**Published:** 2026-04-17

**Authors:** Alireza Dehghan, Karim Abbasi, Mohammad Rasoul Kazemi Najaf Abadi

**Affiliations:** Department of Computer Engineering, Faculty of Technology and Engineering, Salman Farsi University of Kazerun, Kazerun 7319673544, Iran; Mosaheb Institute for Mathematical Research, Kharazmi University, Tehran 1571914911, Iran; Mosaheb Institute for Mathematical Research, Kharazmi University, Tehran 1571914911, Iran

## Abstract

**Motivation:**

Predicting drug–disease associations (DDAs) is essential for efficient drug repurposing. Although graph convolutional networks (GCNs) on heterogeneous drug–disease graphs are state-of-the-art, they often underutilize the rich, multi-modal data available for drugs, such as targets, enzymes, pathways, and chemical substructures.

**Results:**

To address this, we introduce Multi-DDA, a novel framework that systematically integrates these multi-modal drug features into a dedicated learning branch. These enriched drug descriptors are hierarchically combined with the outputs of each graph convolution layer, allowing subsequent layers to selectively refine the most informative node representations. This multi-modal fusion creates more comprehensive drug and disease embeddings. The representations are then processed by a graph attention layer to weigh the importance of different node connections before a final Multi-Layer Perceptron predicts the association matrix. Evaluated on a benchmark dataset of 269 drugs and 598 diseases, Multi-DDA outperforms seven existing methods across key metrics—Area Under the Precision-Recall Curve (AUPR), Area Under the Receiver Operating Characteristic Curve (AUC), and Recall. The significant gains in AUPR and Recall demonstrate its enhanced capability to identify potential DDAs, offering a powerful tool for advancing personalized medicine and drug discovery.

**Availability and implementation:**

The source code for Multi-DDA is freely available at https://github.com/dehghan1401/Multi-DDA

## 1 Introduction

The discovery of effective treatments for diseases is a complex and time-consuming process, often involving extensive research and experimentation (Dehghan *et al.* 2024, Abbasi *et al.* 2025). Identifying potential therapeutic relationships between drugs and diseases is crucial in this process. Predicting drug–disease associations (DDAs) involves using computational methods to identify potential links between drugs and diseases to facilitate the development of new treatments (Kumar Das *et al.* 2021, Masoudi-Sobhanzadeh *et al.* 2021, Zhou *et al.* 2021, Du *et al.* 2022, Zhao *et al.* 2022).

Identifying associations between drugs and diseases is a crucial step in developing new treatments, but it can be costly and time-consuming when relying on traditional laboratory experiments. To address this challenge, researchers have been developing computational methods to accurately predict these associations (Dehghan *et al.* 2023, Rafiei *et al.* 2023).

The human body is a complex system with millions of potential interactions between genes, proteins, and other biological molecules. Diseases are often the result of dysregulation of these interactions, and effective treatments must be able to modulate these interactions to restore normal function. With thousands of approved drugs and countless potential disease targets, the number of possible drug–disease combinations is staggering. Experimental screening of all possible combinations is impractical, making computational methods essential for identifying promising leads (He *et al.* 2025, Su *et al.* 2025, Tang *et al.* 2025).

Several computational approaches have been developed to predict DDAs. These approaches are divided into three important categories, including (Kim *et al.* 2022, Ao *et al.* 2023, Huang *et al.* 2025): (i) Network-based methods: These methods use networks of biological interactions to identify potential DDAs. For example, a network of protein-protein interactions can be used to identify proteins associated with a particular disease, and then drugs targeting those proteins can be identified as potential treatments. (ii) Machine learning-based methods: These methods use machine learning algorithms to identify patterns in large drug and disease information datasets. For example, a machine learning model can be trained on a dataset of known DDAs and then used to predict potential associations for new drugs or diseases. (iii) Text mining-based methods: These methods use natural language processing techniques to extract information from large collections of biomedical texts, such as scientific articles and clinical trial reports (Kang *et al.* 2023).

In recent years, a new wave of approaches has emerged that combines the strengths of network-based methods with the predictive power of machine learning-based methods (Zeng *et al.* 2019, Li *et al.* 2020, Aragh *et al.* 2025). These hybrid approaches leverage the structural information in biological networks and the pattern recognition capabilities of machine learning algorithms to improve the accuracy of DDA predictions. These methods construct a heterogeneous graph using drug–drug, disease–disease, and drug–disease similarity matrices and then apply graph convolutional networks (GCN) with attention layers. However, we have designed a significant enhancement by designing a new branch that aims to learn a stronger representation of drugs (Huang *et al.* 2025). This new representation leverages the Target, Enzyme, Pathway, and Substructure features of drugs. By incorporating these features, we are enriching the drug descriptors with more detailed biochemical and pharmacological information.

The key innovation in our approach is adding this enhanced drug representation to the output of each graph convolution layer. This modification is expected to improve the node descriptors, as it combines the structural information learned from the graph convolutional layers with the biochemical and pharmacological properties of the drugs at three levels. This multi-modal fusion approach enables the method to capture both the relationships between drugs and diseases and the molecular structure of the drugs, leading to improved prediction performance. The expected outcome of the proposed method is to aid in the discovery of new DDAs and facilitate personalized medicine. By combining the strengths of GCNs and convolutional neural networks, the proposed method has the potential to significantly impact pharmaceutical research and development.

## 2 Related work

Zhang *et al.* (2018a, 2018b) introduced a new computational method called Similarity Constrained Matrix Factorization for Drug–Disease Association Prediction (SCMFDD). This method leverages known DDAs, drug features, and disease semantic information to project the relationship into two low-rank spaces, uncovering latent features for drugs and diseases. By incorporating drug feature-based similarities and disease semantic similarity as constraints, SCMFDD considers the problem’s biological context, distinguishing it from classic matrix factorization techniques.

Yu *et al*. (2021) have introduced that utilizes a type of neural network called a GCN to integrate multiple data sources, including known associations between drugs and diseases and similarities between different drugs and diseases. This approach uses a technique called attention to combine the information learned from multiple layers of the network and then uses this integrated information to predict the likelihood of unobserved associations. The results of this approach have been promising, with evaluations showing that it can outperform existing methods in terms of accuracy and ability to identify novel associations. Additionally, a case study has demonstrated the potential of this approach to discover new and previously unknown associations between drugs and diseases. The identification of microRNAs (miRNAs) associated with specific diseases is a crucial step in understanding the development of human diseases, but experimental methods can be time-consuming and labor-intensive. Researchers have turned to computational methods to address this challenge to predict potential miRNA-disease associations. However, existing methods often overlook the important role of genes in mediating these associations and are hindered by sparse data.

Computational drug repositioning can be approached as a recommendation problem, aiming to predict new treatments by completing the unknown entries of a DDA matrix. A bounded nuclear norm regularization (BNNR) method is introduced in (Wang *et al.* 2014), where a balance is struck between approximation error and low-rank properties to manage data noise, and predictions are constrained to a specified range. The BNNR method has been demonstrated to achieve superior prediction accuracy, with a notable improvement in precision, making it particularly valuable for practical drug design applications.

A computational framework based on a heterogeneous network model is introduced for drug repositioning, integrating multi-omics data on diseases, drugs, and targets (Yang *et al.* 2019). The novelty of this method lies in how disease-drug pair strength is calculated through an iterative algorithm on the integrated network. The framework’s superior performance over existing methods is demonstrated through comprehensive experiments, and its practical utility is further validated by case studies. Luo *et al*. (2018) formulated computational drug repositioning as a recommendation system, where the problem is formulated as a low-rank matrix completion task to predict new DDAs. A heterogeneous network integrating drug and disease data is constructed and represented as an adjacency matrix, which is subsequently completed using a fast Singular Value Thresholding (SVT) algorithm to score potential interactions. This Drug Repositioning Recommendation System (DRRS) is shown to achieve higher prediction accuracy than current state-of-the-art methods, a capability validated through comprehensive experiments and case studies.

A new approach has been developed that utilizes multi-task learning to integrate information from both miRNA-disease and gene-disease networks, providing a more comprehensive understanding of the relationships between miRNAs, genes, and diseases (He *et al.* 2023). This approach, called MTLMDA, has been evaluated on a dataset of experimentally validated miRNA-disease associations and has demonstrated superior performance compared to existing methods. Further analysis has shown that the inclusion of gene-disease network information is a key factor in the model’s success, and the approach has been applied to predict miRNA-disease associations for several types of cancer, highlighting its potential for identifying new therapeutic targets.

MeSH (Medical Subject Headings) descriptors of diseases are a standardized vocabulary used to describe and categorize diseases, disorders, and other health-related concepts. MeSH is a controlled vocabulary developed by the National Library of Medicine (NLM) to provide a consistent and accurate way to index and retrieve biomedical literature. By using a controlled set of terms, MeSH enables researchers, clinicians, and other healthcare professionals to efficiently search, organize, and analyze large amounts of data related to diseases and disorders. The use of MeSH descriptors for diseases facilitates the classification of conditions into specific categories, allowing for more precise and targeted searches of the biomedical literature. This, in turn, can help to identify patterns, trends, and relationships between different diseases, ultimately contributing to a better understanding of the underlying causes and mechanisms of various health conditions. The hierarchical structure of MeSH can be represented as a Directed Acyclic Graph (DAG), where diseases are nodes connected by “is-a” or “part-of” relationships (Riseberg *et al.* 2023). This structure allows for a more nuanced calculation of semantic similarity than a flat list, as it accounts for the shared ancestry of disease concepts.

Gao *et al.* (2022) introduce a new approach to finding connections between medications and illnesses by using complex algorithms to analyze similarities between drugs and diseases, allowing researchers to predict new uses for existing medications. They have combined multiple data sources and utilized advanced computational techniques. Wang *et al*. (2021) have introduced a novel approach to achieve this, called CMAF, which leverages ensemble learning to identify potential connections between drugs and diseases. By analyzing known relationships and constructing similarity networks, this method uses a combination of predictive models to determine the likelihood of a drug being effective for a particular disease, even for newly discovered or untested pairings.

## 3 Methods

The proposed approach is explained in this section. First, the dataset is given, then each step is explained in detail.

In the context of this study, the biological and chemical features used to represent drugs are defined as follows:

- **Target:** This refers to the specific biomolecules, primarily proteins, with which a drug interacts to exert its pharmacological effect. These are often disease-associated proteins, such as receptors or enzymes, whose activity is modulated by the drug.
- **Enzyme:** This feature specifically denotes enzymes that are known to be affected by the drug, either through inhibition, induction, or as a substrate. This is a subset of target interactions with a specific functional role.
- **Pathway:** This describes the biological pathways (e.g. metabolic or signaling pathways) in which a drug’s targets are involved. It provides a systems-level view of the drug’s mechanism of action.
- **Substructure:** This refers to the characteristic chemical fragments or functional groups within a drug’s molecular structure. These substructures are derived from the drug’s Simplified Molecular-Input Line-Entry System (SMILES) notation and are critical determinants of its chemical properties and biological activity.

By integrating these multi-modal features, our model aims to capture a more comprehensive representation of a drug’s mechanism, from its atomic-level structure to its system-level effects.

### 3.1 Datasets

The dataset utilized in this study was sourced directly from (Zhang *et al.* 2018a, 2018b, Yu *et al.* 2021), which provides a pre-processed and publicly available collection of DDAs. This dataset was constructed by compiling data from established chemical-disease relationship databases, followed by a rigorous selection process. This process ensured data robustness by including only drugs associated with >10 diseases (i.e. >10) and diseases associated with >10 drugs, resulting in a final set of 18 416 associations between 269 drugs and 598 diseases.

We employed this dataset exactly as provided by the authors of (Zhang *et al.* 2018a, 2018b, Yu *et al.* 2021). Therefore, the features for the 269 drugs (including substructures, targets, enzymes, pathways, and drug–drug interactions) and the MeSH descriptors for the 598 diseases are identical to those in the source. From this main dataset, the subset of 6244 therapeutic associations was also directly utilized for specific analyses. By leveraging this well-established dataset, our study ensures reproducibility and allows for direct comparison with prior work. Detailed information about the Therapeutic dataset (Subset containing ONLY therapeutic relationships) and the Main datasets contains all known drug–disease relationships from the Comparative Toxicogenomics Database (CTD) (Davis *et al.* 2019) is shown in Table 1.

**Table 1:** Summary of therapeutic dataset and main datasets.

Dataset	Drug	Diseases	Known associations	Drug feature				
				Enzyme	Target	Pathway	Substructure	DDI
Therapeutic dataset	269	598	6244	247	623	465	881	2086
Main dataset	269	598	18 416	247	623	465	881	2086

### 3.2 Problem formulation

Given ${\left\{(c^{\left(i\right)},d^{\left(i\right)}),a^{\left(i\right)}\right\}}_{i=1}^{N}$ where $\left(c^{\left(i\right)},d^{\left(i\right)}\right)$ is the $i^{\mathrm{th}}$ drug compound ($c^{\left(i\right)})$ and disease ($d^{\left(i\right)}$) pairs as input, and the $a^{\left(i\right)}$ is its corresponding association with them. Also, N is the number of training samples. It should be noted that each drug compound is represented using multiple modalities (i.e. features), including the SMILES sequence (shown by $c_{s}^{\left(i\right)}$), target (shown by $c_{t}^{\left(i\right)}$), enzyme (shown by $c_{e}^{\left(i\right)}$), drug interaction (shown by $c_{n}^{\left(i\right)}$), pathway (shown by $c_{p}^{\left(i\right)}$) and substructures (shown by $c_{l}^{\left(i\right)}$). In most DDA prediction approaches, the drug–drug similarities matrix and disease-disease similarities matrix are utilized as inputs. In this study, we have utilized them as input, too. The goal is to predict the existence of an association (a link) between a drug node and a disease node in the graph. In other words, the DDA prediction task is framed as a binary classification problem.

#### Drug–drug similarity

As mentioned, a drug is represented by various biological and chemical features, including target, enzyme, drug interaction, pathway, and substructures. The use of multiple feature vectors enables the calculation of various similarity measures between drugs. Certain metrics, such as those that assess the overlap between feature sets (i.e. Jaccard index) or evaluate the cosine of the angle between vector representations (i.e. Cosine similarity), have become widely accepted in the field for quantifying drug similarities (Yu *et al.* 2021, He *et al.* 2023). These measures provide a way to compare and contrast drugs based on their distinct characteristics, which can be useful in a variety of applications, including drug discovery and development.

The Jaccard index is statistically rigorous for biological presence-absence data and is a fundamental choice for gauging similarity and diversity between sample sets (Chung *et al.* 2019). Torab-Miandoab *et al*. (2023) have done a study on drug similarity, specifically found the Jaccard measure to have the best overall performance for predicting drug–drug similarity based on indications and side effects, outperforming other metrics. In disease-disease similarity studies, the Jaccard index is a standard tool for comparing sets of associated genes or pathways, helping to uncover shared molecular mechanisms (Amini-Parikhani *et al.* 2020).

To compute the similarity between two drugs i and j, we concatenate all their feature vectors and calculate the Jaccard index. The drug–drug similarity matrix $S^{c\;}$is defined as follows, where each element $S_{ij}^{c}$ represents the similarity between drug iand drug j:


(1) $$S_{ij}^{c}=\mathrm{Jaccard}({\left|\right|}_{k\in \{t,e,n,p,l}c_{k}^{\left(i\right)},{\left|\right|}_{k\in \{t,e,n,p,l\}}c_{k}^{\left(j\right)})$$

Here, $c_{k}^{\left(i\right)}$ denotes the feature vector for the k-th modality (Target, Enzyme, drug interaction Network, Pathway, or substructure) of the i-th drug. The operator ∥ denotes the vector concatenation operation. The Jaccard index $\mathrm{Jaccard}(x_{i},x_{j})$ between two binary vectors $x_{i}$ and $x_{j}$ is defined as the size of their intersection divided by the size of their union:


(2) $$\mathrm{Jaccard}(x_{i},x_{j})=\frac{\left|x_{i}\cap x_{j}\right|}{\left|x_{i}\cup x_{j}\right|}$$

where ∩ and ∪ show the intersection and union operators, respectively. Also, |.| denotes the cardinality of the set

### 3.3 Disease-disease similarity

The MeSH ontology organizes disease concepts in a hierarchical structure represented as a DAG, denoted as G = (N, E) G = (N, E). Here, NN is the set of nodes, where each node represents a specific disease descriptor, and EE is the set of directed edges representing the semantic relationships between these descriptors. In this DAG, a directed edge from a parent node (a broader disease category, e.g. “Liver Diseases”) to a child node (a more specific disease, e.g. “Hepatitis”) indicates a relationship such as “is-a” (the child is a type of the parent) or “part-of” (the child is a component of the parent).

We have used a similar approach in [21] to compute the similarities between diseases. In this case, the semantic similarity between the disease $d_{i}$ and disease $d_{j}$ is defined as follows:


(3) $$S_{ij}^{d}=\;\frac{\sum_{n\in N(d_{i})\cap N(d_{j})}\left(C_{d_{i}}(n)+C_{d_{j}}(n)\right)}{\sum_{n\in N(d_{i})}C_{d_{i}}(n)+\sum_{n\in N(d_{j})}C_{d_{j}}(n)}$$

where $N(d_{i})$ denotes the set of all ancestor nodes of the disease $d_{i}$ in the MeSH DAG, including $d_{i}$ itself. The term $C_{d}(n)$ represents the contribution of a node n to the semantic value of disease d. It is defined recursively, assigning a higher weight to nodes that are closer to the disease d:


(4) $$C_{d}(n)=\{\begin{array}{cc} 1\; & if\;n=d\\ \underset{n\prime \in N(n)}{\max}\Delta *C_{d}(n^{\prime }) & if\;n\neq d \end{array}$$

where the parameter Δ acts as a discount factor. The rationale for employing distinct similarity measures for drugs and diseases stems from the fundamentally different nature of their available data and the type of relationships we aim to capture. This multi-modal approach allows us to integrate the most relevant biological knowledge for each entity.

- **For Drugs:** The biological features—Target, Enzyme, Pathway, and Substructure—represent explicit, categorical attributes. A drug either interacts with a target or it does not; it contains a substructure, or it does not. The Jaccard index is ideally suited for this type of binary, set-based data as it quantifies similarity based on the overlap of shared features, effectively identifying drugs that operate through common biological mechanisms.
- **For Diseases:** The MeSH ontology provides a hierarchical, semantic structure. Here, the relationship between diseases is not based on shared explicit features but on their conceptual proximity within a defined taxonomy (e.g. “Hepatitis” is a type of “Liver Disease”). The graph-based similarity measure we employ is specifically designed for this context, as it calculates relatedness by measuring the shared ancestry of two diseases within the hierarchical tree. This captures the clinical and phenotypic relatedness that a feature-based method would miss.

By integrating these complementary similarity types—feature-based for drugs and semantic-based for diseases—we construct a richer and more biologically grounded heterogeneous graph. This provides a more comprehensive foundation for the graph neural network to learn from, reflecting the true multi-faceted nature of DDAs.

#### 3.3.1 Knowledge integration

In this study, we aim to integrate multiple types of relationships to comprehensively represent the complex interactions between drugs, diseases, and their underlying mechanisms. To achieve this, we construct a heterogeneous graph shown by $G^{h}=({\left\{c_{i}^{u}\right\}}_{i=1}^{\left|C\right|}\cup {\left\{c_{i}^{d}\right\}}_{i=1}^{\left|D\right|},\;A)$, which encompasses two primary types of nodes: drugs (i.e. denoted by ${\left\{c_{i}^{u}\right\}}_{i=1}^{\left|C\right|})$ and diseases (i.e. denoted by ${\left\{c_{i}^{u}\right\}}_{i=1}^{\left|C\right|}$). The edges in this graph are composed of three distinct types:

1. Drug–disease associations (Known links): These edges connect drugs to diseases using the knowledge provided in the dataset (shown by $A_{\mathrm{known}}\in R^{|C|\times |D|}$). It should be mentioned that for all other pairs of drug diseases (i.e. those that do not exist in the dataset), it is assumed that there is no relation between them.
2. Disease–disease similarities: These edges represent the relationships between diseases based on their shared characteristics (shown by $S^{d}\in R^{|D|\times |D|}$*).* This allows us to utilize the disease-disease similarity matrix as an adjacency matrix to capture the complex interplay between diseases.
3. drug–drug interactions: These edges model the connections between drugs, enabling the identification of drug pairs that tend to co-occur or influence each other’s progression (shown by $S^{c}\in R^{|C|\times |C|}$).

The full adjacency matrix $A\in R^{\left(|C|+|D|)\times (|C|+|D|\right)}$ for the heterogeneous graph is then constructed by combining these three components in a block structure. Given |C| and |D| denote the number of unique drug compounds and the number of unique diseases. In the constructed heterogeneous graph, the number of nodes is |C|+|D|; hence, the adjacency matrix is $A_{\left(|C|+|D|)\times (|C|+|D|\right)}$. The adjacency matrix is defined as follows:


(5) $$A=[\begin{array}{cc} S^{c} & A_{\mathrm{known}}\\ {A_{\mathrm{known}}}^{T} & S^{d} \end{array}]$$

In this formulation:

-
$S^{c}$ is the ∣C∣×∣C∣ drug–drug similarity matrix.
-
$S^{d}$ is the ∣D∣×∣D∣ disease-disease similarity matrix.
-
$A_{\mathrm{known}}$ is the ∣C∣×∣D∣ known DDA matrix, where ∣C∣ is the number of drugs and ∣D∣ is the number of diseases.
- The element $\tilde{A}(c,d)$ indicates the known association between drug cand disease d:
  (6) $$A_{\mathrm{known}}(c,\;d)=\{\begin{array}{cc} a & if\;(c,d)\in D\;\\ 0 & \mathrm{otherwise} \end{array}$$

**Figure 1:**
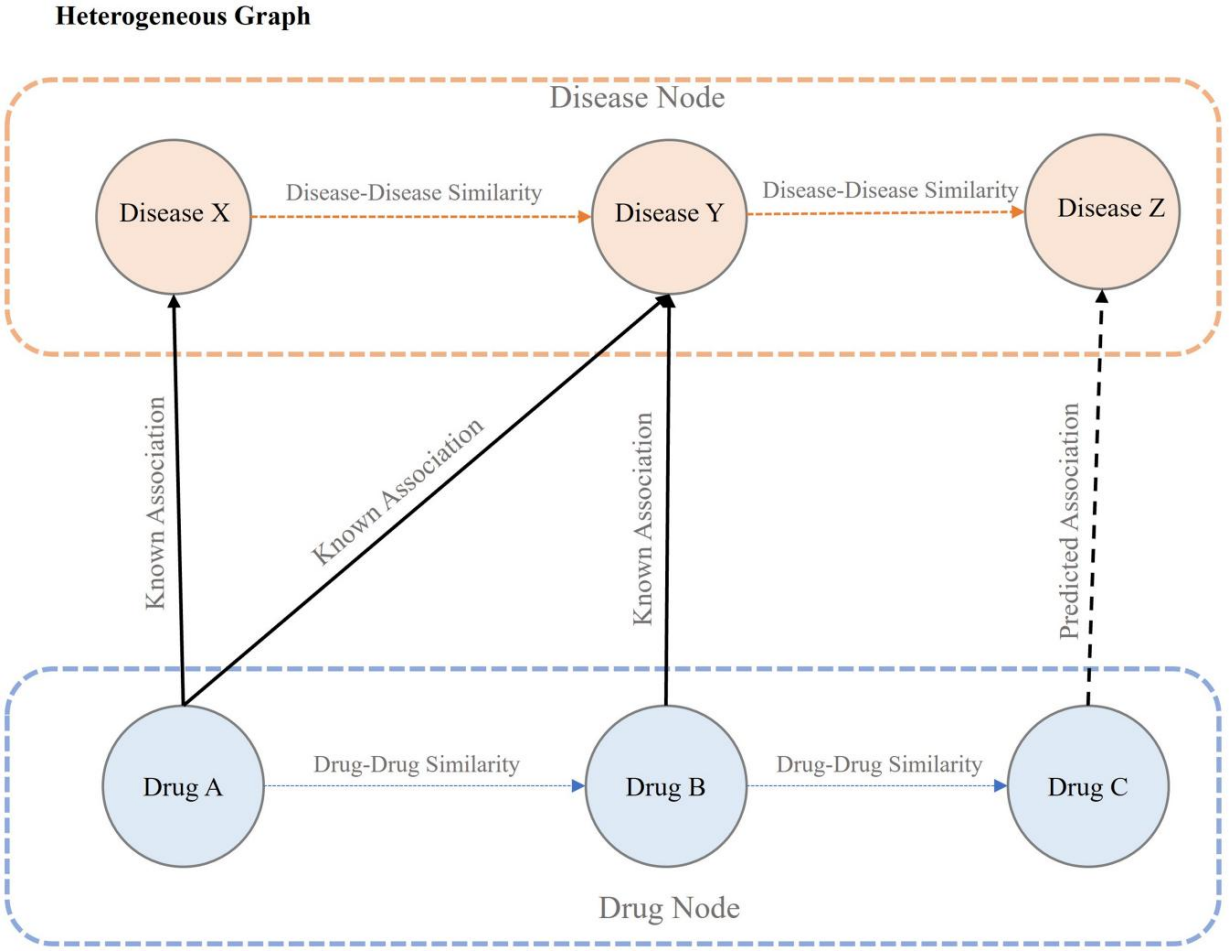
The heterogeneous graph structure connecting drugs and diseases via similarity metrics and known associations.

Figure 1 illustrates the heterogeneous graph that serves as primary input to our Multi-DDA framework, consisting of drug nodes and disease nodes interconnected through three edge categories. The graph encapsulates multiple biological perspectives: drug similarity based on shared targets, enzymes, pathways, and substructures; disease-relatedness through MeSH ontology; and known therapeutic associations from clinical databases. This rich network structure provides the foundational topology for our graph neural network to learn meaningful representations and predict novel drug repositioning opportunities.

### 3.4 Model architecture

**Figure 2:**
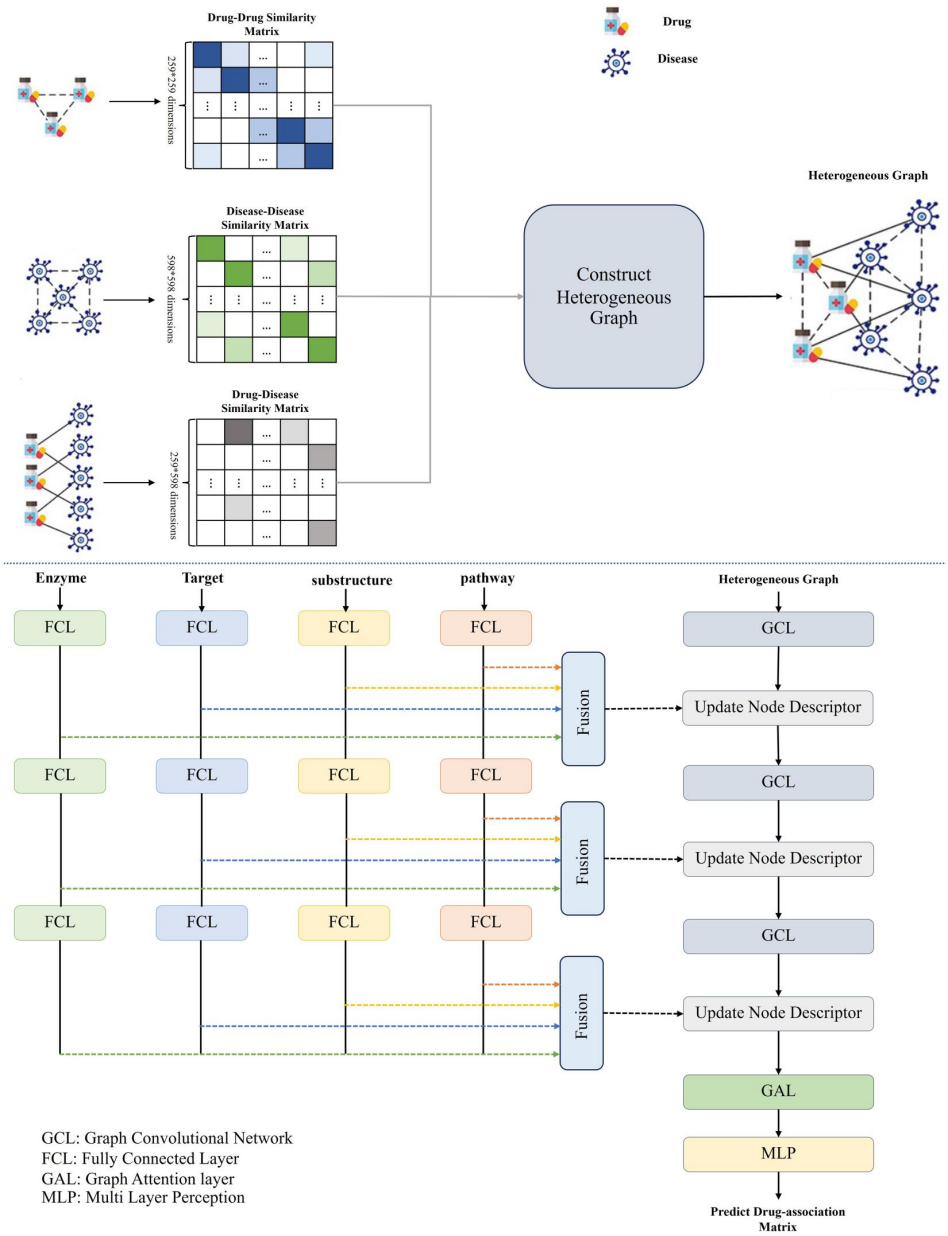
An overview of the multi-DDA.

This section provides the proposed architecture for extracting features from the constructed heterogeneous graph. The overall schematic of the Multi-DDA approach is given in Fig. 2. As it is shown, the proposed model is composed of the graph convolutional layer (GCL), graph attention layer (GAL), and fully connected layer (FCL). The recent work fed the heterogeneous graph of drug diseases into the GCN. To recap, there are some challenges in the recent works, like ignoring the raw sequence of drug compounds and utilizing the provided DDA in the input dataset as an initial representation of the nodes. To cope with these challenges, in the proposed model, not only is the constructed heterogeneous graph used as input, but also the auxiliary knowledge about the drugs, including enzyme, target, pathway, and substructure representation of the drugs, is fed as input to provide more discriminative information to update the drug nodes’ features.

The proposed architecture employs a multi-branch framework to learn a comprehensive representation of drugs by integrating Target, Enzyme, Pathway, and Substructure features. A key aspect of this design is the use of separate projection layers (FCLs) for each feature type. The primary rationale for this design is to grant the model the flexibility to adapt each distinct modality into a shared latent space. The four feature types (Target, Enzyme, Pathway, Substructure) originate from different biological contexts and have unique statistical characteristics.

The subsequent sections provide an in-depth overview of our proposed approach. We begin by introducing the fundamentals of the graph convolutional layer and GAL, followed by a detailed description of how we integrate them with the provided auxiliary knowledge to form our hybrid architecture.

### 3.5 Graph attention networks

Graph Attention Networks, or GATs, are a type of neural network architecture designed to handle graph-structured data (Velickovic *et al.* 2017, Brody *et al.* 2021). They are an extension of traditional GCNs (see Supplementary Section 2, available as supplementary data at *Bioinformatics Advances* online for complete technical details), with the capability to selectively weight the importance of neighboring nodes using an attention mechanism. This allows GATs to learn more nuanced and context-dependent representations of nodes within a graph.

The GAL refines node representations using an attention mechanism to weight the importance of neighbors. The update for the feature of node i at layer l+1 is:


(7) $$h_{i}^{l+1}=\sigma (\frac{1}{K}\sum_{k=1}^{K}\sum_{j\epsilon N_{i}}\alpha_{ij}^{k}W_{g}^{k}h_{j}^{l})$$

where K is the number of independent attention heads, $W^{k}$ is the learnable matrix for the $k^{\mathrm{th}}$ head, and $\alpha_{ij}^{k}$ is the attention coefficient for the $k^{\mathrm{th}}$ head, which is computed as follows:


(8) $$\alpha_{ij}^{k}=\frac{\exp\left(\mathrm{LeakyReLU}(a^{T}[W^{k}h_{i}^{l}||W^{k}h_{j}^{l}])\right)}{\sum_{k\in N_{i}}\exp\left(\mathrm{LeakyReLU}(a^{T}[W^{k}h_{i}^{l}||W^{k}h_{k}^{l}])\right)}$$

### 3.6 Integration module

The proposed architecture is designed to learn a comprehensive representation of drugs by integrating multiple types of features, including Target, Enzyme, Pathway, and Substructure. This is achieved through a multi-branch framework, where each branch is dedicated to learning a representation from a specific feature type. The outputs from these branches are then combined using a weighted sum, allowing the model to learn the importance of each feature type during training. This approach enables the model to capture the complex relationships between different features and leverage their complementary information to improve the accuracy of DDA predictions.

In each branch, a projection layer is used to transform the input features into a lower-dimensional space, reducing the risk of overfitting and improving the efficiency of the model. The projection layer is followed by a ReLU activation function, which introduces non-linearity and enables the model to learn more complex representations. Using ReLU also helps avoid the vanishing gradient problem, allowing the model to learn effectively even with deep architectures. By using a weighted sum to combine the outputs from the different branches, the model can adaptively assign importance to each feature type based on their relevance to the prediction task, leading to more accurate and robust predictions.

The combined representation learned by the proposed architecture is then integrated with the GCN to predict DDAs. The GCN applies graph convolution operations to the integrated representation, learning the structural information and relationships between drugs and diseases. The output of the GCN is then used to predict the likelihood of a DDA. The use of a weighted sum and ReLU activation function in the proposed architecture has been shown to be effective in learning a comprehensive representation of drugs, leading to improved prediction accuracy and robustness.

In the architecture, a dual-branch framework is employed, where graph convolutional layers and FCLs are integrated to extract complementary features from the heterogeneous graph and four other representations of drug compounds. Specifically, the constructed heterogeneous graph is first processed by a graph convolutional layer, which captures the complex relationships and structural information between different nodes. In this case, the initial node representation $H^{0}$ for the first GCL is set to reflect the graph structure, using the association matrix representation is set as follows:


(9) $$H^{0}=[\begin{array}{cc} 0 & \tilde{A}\\ {\tilde{A}}^{T} & 0 \end{array}]$$

This means that this layer tries to learn the structural knowledge of the input graph. In parallel, four other representation drug compounds, including enzyme, target, pathway, and substructure, are fed into FCLs. Then, their output is fused. In this paper, we have used a concatenation layer. Next, it is used to update the drug node descriptor in the output of the graph convolutional layer. In other words, this proposed architecture extracts sequential patterns and features from the molecular representations. Then, the input of the second layer of the graph convolutional layer is updated as follows:


(10) $$h_{i}^{l}=[h_{i}^{l}||o_{i}^{l}]$$

Here, $o_{i}^{l}$ is the output for drug ifrom the fusion of the dedicated fully connected branches for target, enzyme, pathway, and substructure features at level l. The operator ∥ denotes the concatenation operation. This updated descriptor is then used as input for the next graph convolution layer.

By combining the outputs of these two branches to update the node descriptor matrix, the model is able to leverage both the graph-based and sequence-based information to provide more discriminative features for the drug compounds. Next, the convolutional operator propagates this information to all nodes (i.e. disease) in the second GCL. It should be noted that this technique of updating the node descriptor is done on three levels. Through a hierarchical process of representation extraction and node descriptor updating, the subsequent drug representation layer can distill and focus on the most salient and relevant information. After updating the node descriptor in the third level, the learned graph is fed into the GAL. Finally, the output of GAL is passed to MLP to learn the DDA matrix.

## 4 Results

In this section, we assess the efficacy of our proposed model in predicting DDAs, with a focus on evaluating its accuracy, robustness, and potential applications. To evaluate the performance of our predictive model, we utilized a cross-validation technique, specifically a five-fold partitioning of the dataset. The entire set of known DDAs was randomly segmented into five equal-sized subsets, each of which was used as a test set in a rotating manner. The remaining four subsets were used as the training set for each iteration. This process was repeated five times, with each subset serving as the test set once.

This study presents a comprehensive comparison of the Multi-DDA with several state-of-the-art approaches, including BNNR (Yang *et al.* 2019), TL-HGBI (Wang *et al.* 2014), DRRS (Luo *et al.* 2018), deepDR (Zeng *et al.* 2019), NIMCGCN (Li *et al.* 2020), SCMFDD (Zhang *et al.* 2018), and LAGCN (Yu *et al.* 2021). The performance of our method is evaluated against these existing methods to provide a thorough assessment of its effectiveness and identify areas for potential improvement.

The performance of our model was assessed using two primary metrics: the Area Under the Precision-Recall Curve (AUPR) and the Area Under the Receiver Operating Characteristic Curve (AUC). These metrics were chosen for their ability to provide a comprehensive evaluation of our model’s performance, independent of any specific threshold. Additionally, we calculated several secondary metrics, including recall (RE), specificity (SP), accuracy (ACC), precision (PRE), and the F1 score, to provide further insight into our model’s strengths and weaknesses. By using this multi-faceted evaluation approach, we aimed to obtain a thorough understanding of our model’s performance and identify areas for potential improvement.

To optimize model performance, we conducted a hyperparameter search. The learning rate was tuned from {0.0001, 0.0005, 0.001, 0.005, 0.01} and the embedding dimension from {64, 128, 256}, while the number of layers was fixed. Training was consistently run for 10 000 iterations.

### 4.1 Ablation study

We conducted an ablation study to comprehensively evaluate each component’s contribution in our proposed approach. Specifically, we investigate the impact of each of the four node representation types—enzyme, target, pathway, and substructure—on the performance of our model. By systematically removing one representation type at a time, we assessed how each type’s absence affects our model’s overall performance.

We created four variants of our model, each with one of the representation types removed:

- **Enzyme Representation**: In this case, only the enzyme representation from the input features is used to update the drug node descriptors.
- **Target Representation**: It utilizes only the target representation from the input features to update the drug node descriptors.
- **Pathway Representation**: We have used only the pathway representation from the input features to update the drug node descriptors.
- **Substructure Representation**: Only the substructure representation from the input features is utilized to update the drug node descriptors.

We then trained and evaluated each of these variants using the same dataset and evaluation metrics as our full model. By comparing the performance of each variant to the full model, we can quantify the contribution of each representation type to the overall performance of our approach.

This ablation study allows us to identify which representation types are most critical to the success of our model and provide insights into how each type of information contributes to the learning of effective node descriptors for drugs. The obtained results are given in Table 2. As shown, the full model, which utilized the four representations of the drugs, achieves better performance in five out of six performance measures. Also, by comparing the four representations, it is found that the substructure representation performs better in all measures compared to the enzyme, target, substructure, and pathway representations.

**Table 2:** The results of the ablation study, which show how each of the drug representation features impacts the final performance of the Multi-DDA.

Method	AUPR	AUC	RE	PRE	SP	ACC	F1
Enzyme rep.	0.3289	0.8799	0.3842	0.3083	0.9698	0.9711	0.3426
Target rep.	0.3321	0.8875	0.3805	0.3024	0.9716	0.9706	0.3367
Substructure rep.	0.3397	0.8908	0.3967	0.3294	0.9791	**0.9836**	0.3610
Pathway rep.	0.3347	0.8894	0.3943	0.3243	0.9771	0.9807	0.3568
**Full Model**	**0.3542**	**0.8941**	**0.4012**	**0.3434**	**0.9801**	0.9824	**0.3702**

Performance metrics are: AUPR, area under the precision-recall curve; AUC, area under the ROC curve; RE, recall; PRE, precision; SP, specificity; ACC, accuracy; F1, F1-score. Values in bold represent the top score for each performance metric across the ablation variants.

### 4.2 Comparison with SOTA

To ensure a fair evaluation, we compared the performance of our proposed Multi-DDA model against seven state-of-the-art methods: BNNR (Yang *et al.* 2019), TL-HGBI (Wang *et al.* 2014), DRRS (Luo *et al.* 2018), deepDR (Zeng *et al.* 2019), NIMCGCN (Li *et al.* 2020), SCMFDD (Zhang *et al.* 2018), and LAGCN (Yu *et al.* 2021). Crucially, this comparison was conducted using the same dataset and the same five-fold cross-validation setup for all methods. This controlled experimental design guarantees that the performance differences observed are attributable to the models’ capabilities rather than variations in the underlying data, thereby allowing for a direct and equitable assessment.

The comprehensive results are presented in Table 3. As shown, the proposed Multi-DDA method achieves superior performance across all six evaluation metrics. Specifically, Multi-DDA attains a 3.74% relative improvement in AUPR and a 4.12% improvement in Recall compared to the next best method, LAGCN. The enhanced performance in these particular metrics indicates that our model is more effective at identifying true positive associations, a critical capability for tasks like drug repositioning in personalized medicine. The consistent outperformance across all metrics underscores the advantage of integrating multi-modal drug representations with a hybrid graph convolutional architecture.

**Table 3:** The comparison of the Multi-DDA with seven state-of-the-art approaches on the same benchmark dataset.

Method	AUPR	AUC	RE	SP	ACC	F1	*P*-value
BNNR (Yang *et al.* 2019)	0.2262	0.8567	0.3403	0.9738	0.9578	0.2894	2.1 × 10^−2^
TL-HGBI (Wang *et al.* 2014)	0.0665	0.7029	0.2545	0.9284	0.9114	0.1266	7 × 10^−3^
DRRS (Luo *et al.* 2018)	0.1321	0.8429	0.3267	0.9468	0.9324	0.2178	2.2 × 10^−2^
deepDR (Zeng *et al.* 2019)	0.1353	0.8211	0.2959	0.9567	0.9400	0.1991	1.9 × 10^−2^
NIMCGCN (Li *et al.* 2020)	0.2002	0.8533	0.3083	0.9739	0.9572	0.2661	2.7 × 10^−2^
SCMFDD (Zhang *et al.* 2018)	0.2659	0.8727	0.3430	0.9783	0.9623	0.3143	2.5 × 10^−2^
LAGCN (Yu *et al.* 2021)	0.3168	0.8750	0.3600	0.9760	0.9605	0.3150	8 × 10^−3^
Multi-DDA	**0.3542**	**0.8941**	**0.4012**	**0.9801**	**0.9824**	**0.3702**	

Performance metrics are: AUPR, area under the precision-recall curve; AUC, area under the ROC curve; RE, recall; SP, specificity; ACC, accuracy; F1, F1-score. Values in bold represent the top score for each performance metric across the seven state-of-the-art approaches.

The observed discrepancy between the AUPR and the AUC across all methods, including ours, is a characteristic result of class imbalance. In our dataset, the number of known associations (positive links) is vastly outnumbered by the number of unknown/non-associated pairs (negative links). The ROC curve and its AUC metric can be overly optimistic in such scenarios, as the high true negative rate (specificity) easily inflates the score. In contrast, the Precision-Recall curve and AUPR are more sensitive to the performance on the positive class and provide a more realistic assessment of a model’s ability to identify the rare, true associations amidst a majority of negatives. Therefore, while the high AUC values indicate good overall ranking ability, the lower and more variable AUPR values highlight the intrinsic difficulty of the prediction task. The significant improvement of Multi-DDA in AUPR (0.3542) over the next best method, LAGCN (0.3168), is thus a strong indicator of its superior performance in this imbalanced setting.

Based on comprehensive paired t-tests comparing the proposed Multi-DDA method against seven existing approaches across six performance metrics (AUPR, AUC, RE, SP, ACC, F1), our method demonstrates statistically significant superiority over all benchmark methods. The analysis reveals consistent and meaningful performance improvements, with all comparisons yielding statistically significant *P*-values (*P* < .05), ranging from 7 × 10^−^³ to 2.7 × 10^−2^. Multi-DDA achieved the highest scores across all evaluation metrics, particularly excelling in F1-score (0.3702), accuracy (0.9824), and recall (0.4012), while maintaining strong performance in specificity (0.9801) and AUC (0.8941). The most pronounced statistical significance was observed against TL-HGBI (*P* = 7 × 10^−^³) and LAGCN (*P* = 8 × 10^−^³), confirming that Multi-DDA represents a substantial advancement over current state-of-the-art methods in DDA prediction.

### 4.3 The impact of the different fractions of known associations

**Figure 3:**
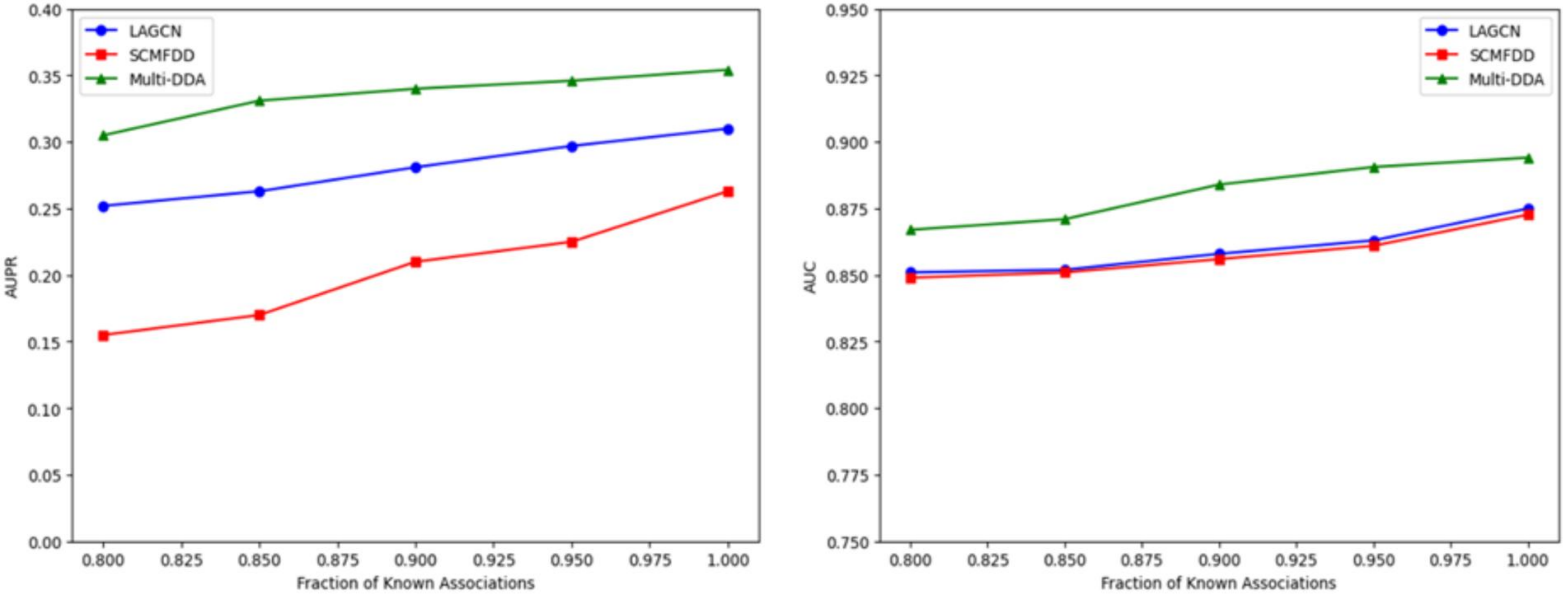
Comparison of the multi-DDA with LAGCN and SCMFDD in the different fractions of known associations.

The impact of the different fractions of known associations on the performance of our model was investigated to assess its robustness and generalizability. We varied the percentage of known associations used for training, ranging from 80% to 100%, and evaluated the model’s performance on the remaining unseen data. Our results showed that the model’s performance improved significantly as the fraction of known associations increased, with the AUPR increasing from 0.3 at 80% known associations to 0.35 at 100% known associations. This suggests that the model is able to effectively leverage the information contained in the known associations to improve its predictions and that the quality of the predictions improves as more information is available. However, even with limited known associations (80%), the model still achieved a respectable AUPR of 0.86, indicating that it is able to generalize well to unseen data and make accurate predictions. These findings have important implications for the application of our model in real-world scenarios, where the availability of known associations may be limited, and demonstrate the potential of our approach to facilitate the discovery of new DDAs (See Fig. 3).

### 4.4 Predicting novel associations

The objective of our model is to uncover new potential relationships between drugs and diseases. To achieve this, we utilize a comprehensive dataset of known DDAs to train our model. The trained model is then employed to predict novel associations that have not been previously established. The prediction process involves analyzing the complex interactions between drugs and diseases, taking into account various factors that influence their relationships. Our model generates a list of predicted associations, ranked according to their confidence scores, which reflect the strength of the predicted relationships.

To identify the most promising candidates for further investigation, we select the ten top-ranked predictions and present them in Table 4. This table provides a summary of the predicted associations, including the involved drugs and diseases. The table also includes information on whether existing evidence in the literature supports each predicted association. This is indicated by the presence of a reference number or “NA” (not available) if no evidence was found. As shown, six out of the ten predicted associations have some evidence in the literature, which suggests that the proposed method can identify potential associations supported by existing research. Prednisone is a corticosteroid medication that is commonly used to treat a variety of conditions, including inflammatory disorders and autoimmune diseases. While it is not typically used as a primary treatment for epilepsy, there is some evidence that corticosteroids like Prednisone may be effective in reducing the frequency and severity of seizures in some individuals with epilepsy. This is thought to be due to the anti-inflammatory properties of corticosteroids, which may help reduce the brain’s inflammation and swelling that can contribute to seizure activity.

**Table 4:** Top ten predicted DDAs by the proposed method.

Drug	Disease	Score	Evidence
Prednisone	Epilepsy	0.96	(Sinclair 2003)
vinorelbine	Anemia, Hemolytic, Autoimmune	0.95	NA
vinorelbine	Lung Injury	0.95	(Tanvetyanon *et al.* 2006)
Toremifene Citrate	Anemia, Hemolytic, Autoimmune	0.93	NA
Biperiden	Endomyocardial fibrosis	0.93	NA
acetylcholine chloride	Splenomegaly	0.93	NA
Midazolam	Migraine disorders	0.96	(Amini-Parikhani *et al.* 2020)
Clozapine	Hyperhidrosis	0.95	(Szymanski *et al.* 1991)
Sirolimus	Anemia, Hemolytic, Autoimmune	0.95	(Bride *et al.* 2016, Zhang *et al.* 2023)
Mebendazole	Anemia, Hemolytic, Autoimmune	0.95	(Girelli *et al.* 1993)

## 5 Discussion

The proposed Multi-DDA framework demonstrates the significant advantage of integrating multi-modal drug features within a hybrid GCN for DDA prediction. Our core innovation—a dual-branch architecture that hierarchically fuses graph-based structural information with rich, biochemical drug descriptors (targets, enzymes, pathways, and substructures)—enables the learning of more comprehensive node representations. This approach directly addresses a key limitation of existing methods that underutilize available pharmacological data.

The model’s effectiveness is confirmed through rigorous evaluation. The ablation study not only validated the contribution of each feature modality but also revealed the chemical substructure as the most influential single representation, highlighting the primary role of a drug’s molecular makeup. Multi-DDA achieved state-of-the-art performance, outperforming seven benchmark methods across all metrics. Crucially, its superior performance in AUPR and Recall was statistically significant (*P*-value <.05 against the best baseline, LAGCN), underscoring an enhanced capability to identify true positive associations—a critical factor for drug repurposing. The model’s practical utility is further evidenced by its ability to predict novel associations, with six of the top ten predictions corroborated by existing literature.

Despite these strengths, we acknowledge limitations that guide our future work. An error analysis indicated that challenges remain in predicting associations for rare diseases with sparse data and for highly polypharmacological drugs. Future efforts will focus on incorporating additional data modalities (e.g. gene expression), refining the graph construction process, and enhancing the model’s explainability to provide deeper biological insights and greater clinical translatability.

## 6 Conclusion

In this study, we introduced Multi-DDA, a novel computational framework for predicting DDAs. The key contribution of our work is the development of a hybrid architecture that effectively integrates multi-modal drug representations with the structural information of a heterogeneous drug–disease graph. Specifically, our model leverages detailed drug features—including targets, enzymes, pathways, and chemical substructures—within a dedicated learning branch. These enriched representations are hierarchically combined with the outputs of graph convolutional layers, allowing the model to learn more comprehensive and discriminative node descriptors.

Our extensive experimental results demonstrate that Multi-DDA significantly outperforms several state-of-the-art methods across all key evaluation metrics, particularly in AUPR and Recall. This underscores its enhanced capability to identify true positive associations, a critical factor for drug repurposing. The ablation study confirmed the importance of each feature modality, with the substructure information proving to be the most influential. Furthermore, the model’s ability to predict novel associations with supporting literature evidence validates its potential as a powerful tool for accelerating drug discovery and personalized medicine. Future work will focus on incorporating additional biological data and exploring the model’s application to predicting specific treatment outcomes.

While the literature validation for several top predictions is encouraging, a limitation of this study is the lack of formal validation by clinical domain experts. The novel predictions presented here, particularly those without existing literature evidence, should therefore be considered as high-confidence computational hypotheses. Future work will focus on collaborating with medical professionals to conduct a systematic expert review to further assess the clinical plausibility and priority of these candidate DDAs.

## Supplementary Material

vbag034_Supplementary_Data

## Data Availability

The datasets were compiled from the following public domain sources: Drug–disease associations: The CTD (http://ctdbase.org/) Drug features: DrugBank (https://www.drugbank.ca/) Disease MeSH descriptors: The MeSH database (https://meshb.nlm.nih.gov/)
