# Supplementary material for: Multi-DDA: drug–disease association prediction using a hybrid graph convolutional network with multi-modal drug representations
Source: Bioinform Adv. 2026 Apr 17;6(1):vbag034. doi: 10.1093/bioadv/vbag034 (PMC13130202; doi:10.1093/bioadv/vbag034)
Supplement: vbag034_Supplementary_Data [file vbag034_supplementary_data.docx]

**Supplementary Data**

**Multi-DDA: Drug-Disease Association Prediction using a Hybrid Graph Convolutional Network with Multi-modal Drug Representations**

**Alireza Dehghan^1, *^, Karim Abbasi^2, *^, Mohammad Rasoul Kazemi Najaf Abadi^2^**

^1^Department of Computer Engineering, Faculty of Technology and Engineering, Salman Farsi University of Kazerun, Kazerun, Iran.

^2^Mosaheb Institute for Mathematical Research, Kharazmi University, Tehran, Iran.

**S.1. Top 100 novel predicted drug-disease associations**

A comprehensive list of the top 100 novel drug-disease associations predicted by our model, ranked by prediction confidence scores, is provided in Table S-1. These predictions represent promising candidates for drug repurposing and include both established therapeutic relationships and novel associations that warrant experimental validation. It enables researchers to explore specific drug-disease pairs of interest and facilitates hypothesis generation for future pharmacological studies.

Table S-1: The top 100 novel drug-disease associations predicted by our model

| Drug | Disease |
| --- | --- |
| Prednisone | Epilepsy |
| vinorelbine | Anemia, Hemolytic, Autoimmune |
| vinorelbine | Lung Injury |
| Toremifene Citrate | Anemia, Hemolytic, Autoimmune |
| Biperiden | Endomyocardial fibrosis |
| acetylcholine chloride | Splenomegaly |
| Midazolam | Migraine disorders |
| Clozapine | Hyperhidrosis |
| Sirolimus | Anemia, Hemolytic, Autoimmune |
| Mebendazole | Anemia, Hemolytic, Autoimmune |
| Bleomycin | Brain Neoplasms |
| Levamisole | Hepatitis |
| Bleomycin | Heart Failure |
| Norepinephrine | Hypertension |
| Hydroxychloroquine | Seizures |
| Methotrexate | Edema |
| Clonidine | Brain Neoplasms |
| Glutamine | Seizures |
| Tetracycline | Hyperthyroidism |
| Levamisole | Diabetes Mellitus |
| Oxycodone | Neoplasms |
| Erythromycin | Hyperthyroidism |
| Guanethidine | Brain Neoplasms |
| Venlafaxine | Hypertension |
| Hydroxychloroquine | Pancreatitis |
| Levamisole | Psychotic Disorders |
| Tamoxifen | Vomiting |
| Zolpidem | Asthma |
| Fluoxetine | Diabetes Mellitus |
| Duloxetine | Neoplasms |
| Phenytoin | Schizophrenia |
| Methotrexate | Depressive Disorder |
| Risperidone | Brain Neoplasms |
| Quetiapine | Fibrosis |
| Diltiazem | Headache |
| Bleomycin | Pain |
| Diazepam | Neoplasms |
| Ethanol | Hypotension |
| Vorinostat | Neoplasms |
| Oxycodone | Substance-Related Disorders |
| Prazosin | Hypertension |
| Thiamine | Edema |
| Triamcinolone | Hepatitis |
| Phenylpropanolamine | Hypertension |
| Pentoxifylline | Hyperthyroidism |
| Pentoxifylline | Stroke |
| Acetaminophen | Myocardial Infarction |
| Memantine | Pain |
| Sunitinib | Heart Diseases |
| Methotrexate | Neoplasms |
| Aminocaproic Acid | HIV Infections |
| Methotrexate | Doxorubicin |
| Oxycodone | Hypertension |
| Norepinephrine | Diabetes Mellitus, Type 2 |
| Flutamide | Thrombosis |
| Vorinostat | Colonic Neoplasms |
| Tolterodine | Pancreatitis |
| Romidepsin | Diabetes Mellitus, Type 2 |
| Isoniazid | Diabetes Mellitus, Type 2 |
| Etoposide | Hyperthyroidism |
| Nitrofurantoin | Edema |
| Tamoxifen | Nausea |
| Phenylpropanolamine | Hypotension |
| Zaleplon | Myocardial Infarction |
| Levamisole | Stroke |
| Phenelzine | Colonic Neoplasms |
| Guanfacine | Brain Neoplasms |
| Sorafenib | Fibrosis |
| Tolterodine | Drug-Related Side Effects and Adverse Reactions |
| Romidepsin | Carcinoma, Hepatocellular |
| Sertraline | Asthma |
| Trazodone | Tremor |
| Guanfacine | Pancreatitis |
| Desipramine | Carcinoma, Renal Cell |
| Paroxetine | Depressive Disorder |
| Doxepin | Carcinoma, Non-Small-Cell Lung |
| Chlorpromazine | Hypertension |
| Furosemide | Heart Diseases |
| Guanfacine | Vision Disorders |
| Clonidine | Diabetes Mellitus, Type 2 |
| Sertraline | Aortic Aneurysm |
| Aripiprazole | Hearing Loss |
| Duloxetine | Myocardial Infarction |
| Tolvaptan | Metabolic Syndrome |
| Risperidone | Renal Insufficiency |
| Tolvaptan | Nervous System Diseases |
| Lurasidone | Liver Diseases |
| Temsirolimus | Stroke |
| Fluoxetine | Dyskinesia, Drug-Induced |
| Sulfasalazine | Psychotic Disorders |
| Amiodarone | Pancreatitis |
| Guanfacine | Stroke |
| Imipramine | Neoplasms |
| Pentamidine | Neoplasms |
| Hydrochlorothiazide | Myocardial Infarction |
| Prazosin | Purpura |
| Fluphenazine | Cataract |
| Hydroxychloroquine | Neoplasm Metastasis |
| Piperazines | Doxorubicin |

## S.2. Graph Convolutional Layer

The graph convolutional layer is a type of neural network layer designed to process data represented as graphs. A graph is a non-Euclidean data structure consisting of nodes (also known as vertices) connected by edges. These layers are inspired by traditional convolutional neural networks (CNNs), which are commonly used for image and signal processing tasks.

Unlike CNNs, which operate on grid-like structures, graph convolutional layers are designed to handle the complexities of graph-structured data. They aim to learn meaningful representations of nodes and edges in the graph by aggregating information from neighboring nodes.

The graph convolutional layer works by applying a set of learnable filters to the graph nodes. Each filter computes a weighted sum of the node features and the features of its neighboring nodes. The weights are learned during training and represent the importance of each neighboring node in the aggregation process.

Given $H$ as a node descriptor matrix, where each row represents the description of a node. As mentioned, the graph convolutional layer aims to update the representation of the nodes using its neighbors. In other words, the matrix $H$ is updated in each convolutional layer as follows:

| $H^{l+1}=\sigma\left( D^{-1/2}AD^{-1/2}H^{l}W_{g}^{l} \right)$ | (s-1) |
| --- | --- |

In this equation

- $H^{l}\in\mathbb{R}^{(\mid C\mid+\mid D\mid)\times F}$is the node feature matrix at layer $l$, where $F$is the feature dimension.
- $\hat{A}=A+I$is the adjacency matrix $A$with added self-connections (represented by the identity matrix $I$), which allows nodes to include their own features in the aggregation.
- $\hat{D}$is the diagonal degree matrix of $\hat{A}$, where $\hat{D}_{ii}=\sum_{j} \hat{A}_{ij}$.
- $W_{g}^{l}$is the layer-specific trainable weight matrix.
- $\sigma(\cdot)$denotes a non-linear activation function, such as ReLU.
